# Degradation of HIF-1alpha under Hypoxia Combined with Induction of Hsp90 Polyubiquitination in Cancer Cells by Hypericin: a Unique Cancer Therapy

**DOI:** 10.1371/journal.pone.0022849

**Published:** 2011-09-19

**Authors:** Tilda Barliya, Mathilda Mandel, Tami Livnat, Dov Weinberger, Gad Lavie

**Affiliations:** 1 The Blood Center, Sheba Medical Center, Ramat-Gan, Israel; 2 Department of Ophthalmology, Beilinson Medical Center, Petah-Tiqva, Israel; 3 Institute of Hematology, Sheba Medical Center, Ramat-Gan, Israel; 4 Department of Ophthalmology, Sackler School of Medicine, Tel-Aviv University, Tel-Aviv, Israel; 5 Department of Cellular and Developmental Biology, Sackler School of Medicine, Tel-Aviv University, Tel-Aviv, Israel; University of Delhi, India

## Abstract

The perihydroxylated perylene quinone hypericin has been reported to possess potent anti-metastatic and antiangiogenic activities, generated by targeting diverse crossroads of cancer-promoting processes via unique mechanisms. Hypericin is the only known exogenous reagent that can induce forced poly-ubiquitination and accelerated degradation of heat shock protein 90 (Hsp90) in cancer cells. Hsp90 client proteins are thereby destabilized and rapidly degraded. Hsp70 client proteins may potentially be also affected via preventing formation of hsp90-hsp70 intermediate complexes. We show here that hypericin also induces enhanced degradation of hypoxia-inducible factor 1α (HIF-1α) in two human tumor cell lines, U87-MG glioblastoma and RCC-C2VHL−/− renal cell carcinoma and in the non-malignant ARPE19 retinal pigment epithelial cell line. The hypericin-accelerated turnover of HIF-1α, the regulatory precursor of the HIF-1 transcription factor which promotes hypoxic stress and angiogenic responses, overcomes the physiologic HIF-1α protein stabilization which occurs in hypoxic cells. The hypericin effect also eliminates the high HIF-1α levels expressed constitutively in the von-Hippel Lindau protein (pVHL)-deficient RCC-C2VHL−/− renal cell carcinoma cell line. Unlike the normal ubiquitin-proteasome pathway-dependent turnover of HIF-α proteins which occurs in normoxia, the hypericin-induced HIF-1α catabolism can occur independently of cellular oxygen levels or pVHL-promoted ubiquitin ligation of HIF-1α. It is mediated by lysosomal cathepsin-B enzymes with cathepsin-B activity being optimized in the cells through hypericin-mediated reduction in intracellular pH. Our findings suggest that hypericin may potentially be useful in preventing growth of tumors in which HIF-1α plays pivotal roles, and in pVHL ablated tumor cells such as renal cell carcinoma through elimination of elevated HIF-1α contents in these cells, scaling down the excessive angiogenesis which characterizes these tumors.

## Introduction

Formation of tumor metastases by disseminating cancer cells and their explosive growth remains the most prevalent cause for cancer treatment failure and death. Tumor cells remodel the extracellular matrix, modify cell adhesion properties, invade surrounding tissues and transmigrate to distal organs to form metastatic foci. Developing foci generate hypoxia and a need for neoangiogenesis to support growth. Hypoxia stabilizes the stress response precursor HIF-1α [1], leading to its translocation to the nucleus via an hsp90 dependent process [2], [3] and heterodimerization with HIF-1β, generating the functional HIF-1 transcription factor. HIF-1 promotes transcription of ∼100 stress response target proteins including VEGF. VEGF stimulates increased expression of its primary receptor VEGFR2. The VEGF-VEGFR2 complexes which form require association with hsp90 to activate the downstream signaling that initiates the neoangiogenic cascade, [4] and activates the integrin-focal adhesion kinase (FAK)-Src signaling complex. Both FAK and Src are also hsp90 client proteins, requiring association with this chaperone for maintaining their functional conformations [5], [6]. These functions include formation of focal adhesions associated with an F-actin contractile apparatus that are linked to the cell membrane and activate the migration machinery via interaction with the extracellular matrix [7]. Thus, Hsp90 inhibition can disrupt several sites in angiogenic and cell dispersion signaling cascades and interfere with tumor progression.

The marked increases in HIF-1α content that occur in many tumor types implicate HIF-1 in promoting oncogenesis. Tumor progression is accelerated via heterogeneous mechanisms including dysfunctional/deleted VHL gene in renal cell carcinoma and hemangioblastoma [8], inactivated IDH1 gene in glioblastoma [9], mutations in mitochondrial succinic dehydrogenases in paraganglioma, and others [10]. Indeed, elevated intratumoral HIF-1α (or HIF-2α) are associated with accelerated patient mortality, evident from retrospective immunohistochemical analyses of paraffin embedded biopsy sections from various tumors [11]. It is currently accepted that diminishing tumoral HIF-1α levels may encompass important clinical benefits, spurring intensive searches for small molecule inhibitors of HIF-1α.

Reagents with diverse activities capable of interfering with tumor cell proliferation, migration and neoangiogenic signaling are likely to more effectively inhibit formation of metastases and benefit cancer patients. One such potentially promising reagent is the perihydroxylated perylene quinone - hypericin. We found that hypericin effectively inhibits formation of metastases by murine breast and squamous cell carcinoma tumors *in vivo* [12], apparently by interfering with signaling pathways that promote angiogenesis [13] and tumor cell proliferation [14]. The common denominator linking these different activities is a unique ability of hypericin to act as exogenous inducer of forced poly-ubiquitination of heat shock protein 90 (Hsp90), destabilizing and rapidly degrading a plethora of hsp90-client proteins [14].

Here we report that hypericin can degrade HIF-1α in cells via a unique hypoxia and proteasome independent mechanism. Although HIF-1α is an hsp90 client protein [15] degraded by other hsp90 inhibitors [16], the hypericin-induced HIF-1α catabolism appears to involve a unique lysosomal cathepsin-B dependent mechanism, activated in a reduced intracellular pH environment. We also show that the angiogenic signaling cascade can be affected by hypericin at multiple sites, rendering this molecule potentially promising in anti cancer therapy.

## Results

### Forced HIF-1α degradation under hypoxia by cell treatment with hypericin

**Figure 1:**
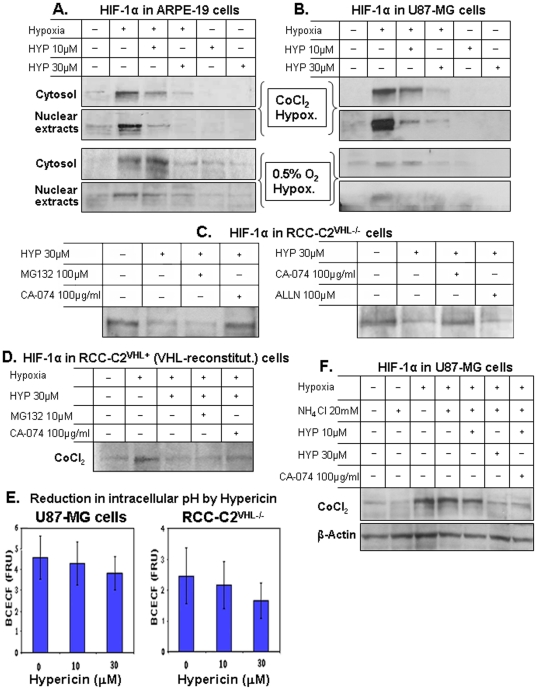
Enhancement of HIF-1α turnover under hypoxic conditions in cells exposed to hypericin. [A] ARPE19, [B] U87-MG, [C] RCC-C2^VHL−/−^ and [D]. RCC-C2^VHL+^ (VHL gene reconstituted) cell lines were exposed to 10 and 30 µM hypericin for 72 hrs. The cultures were subjected to hypoxic conditions with 150 µM CoCl_2_ (A & B, upper panel doublets) and with a low oxygen atmosphere (0.5% O_2_, 94.5% N_2_ and 5% CO_2_) (A & B, lower panel doublets) for the last 6 hours of treatment. Hypericin caused degradation of HIF-1α. The mediator of HIF-1α proteolytic cleavage was characterized by inhibition of HIF-1α degradation with the CA-074 cathepsin B inhibitor, with MG-132 and with ALLN proteasomal calpain inhibitors, administered to the cultures for the last 6 hours of incubation with the cells. Cytosolic extracts and nuclear extracts were prepared, separated on SDS-PAGE and Western blots developed with anti-HIF-1α. Equal loading was confirmed with β-Actin. [E]. Effects of hypericin on the intracellular pH of U87-MG and C2^VHL−/−^ cells. Quantitative BCECF, pH-dependent fluorescence was measured spectrofluorimetrically (FRU denotes Fluorescence Relative Units). [F]. Analysis of the role of hypericin-mediated intracellular pH reduction in promoting HIF-1α turnover under CoCl_2_ hypoxia, determined following cytoplasmic alkalinization with 20 mM NH_4_Cl applied during the last 6 hrs of incubation. Cytoplasmic alkalinization diminished the hypericin-induced HIF-1α turnover under CoCl_2_ hypoxia.

Aiming to decipher the mechanism for the anti-angiogenic activity of hypericin [13], we examined whether hypericin affects HIF-1α adaptive stabilization, which occurs under hypoxia in the absence of proline and asparagine hydroxylation [1] in three human cell lines: U87-MG glioblastoma cells, RCC-C2^VHL−/−^ (C2^VHL−/−^) renal carcinoma cells deficient in pVHL, and ARPE-19 retinal pigment epithelial cells. The cells were first exposed to hypericin for 72 hrs, the time required for optimal hypericin effects to develop and hypoxia generated chemically with CoCl_2_ and with a low oxygen atmosphere (0.5% O_2_, 5% CO_2_ and 94.5% N_2_) for the last 6 hours of treatment to prevent hypoxic cytotoxicity. HIF-1α levels were analyzed in cytosolic and nuclear fractions by Western blots. The results, Fig. 1 show that exposure to 10 µM and 30 µM of hypericin effectively reduced the hypoxia-induced increases in HIF-1α levels in a hypericin dose dependent manner in ARPE-19 cells (Fig. 1A) and in U87-MG cells (Fig. 1B). Reductions in HIF-1α were more pronounced in the nuclear fractions of both cell lines, particularly when hypoxia was induced chemically with CoCl_2_.

To determine how hypericin enhances HIF-1α degradation we used the VHL gene-deleted C2^VHL−/−^ cell line. pVHL, the substrate recognition and binding module of an E-3 ubiquitin ligase complex binds HIF-1α following prolyl hydroxylation on the HIF-1α prolyl-hydroxylase domain protein-2 [17], thereby ubiquitinating HIF-1α and mediating the proteasomal degradation of this stress-response transcription factor precursor [18]. pVHL deficiency disrupts this oxygen-dependent degradation response, leading to constitutive accumulation of HIF-1α. We examined the HIF-1α content in C2^VHL−/−^ cells following 72 hr treatments with hypericin. The constitutively high HIF-1α content in C2^VHL−/−^ cells decreased following exposure to hypericin (Fig. 1C exhibits) in a manner similar to the reductions in HIF-1α observed in U87-MG and ARPE-19 cells treated with hypericin under hypoxia. HIF-1α elimination by hypericin in C2^VHL−/−^ cells occurred independently of pVHL and was found to be insensitive to inhibition with MG132 or ALLN proteasomal calpain inhibitors (Fig. 1C), thus excluding involvement of the ubiquitin-proteasome pathway in this process. HIF-1α degradation by hypericin was, however effectively inhibited by the cathepsin-B inhibitor CA-074 in C2^VHL−/−^ cells (Fig. 1C) and unaffected by CLi-III, a cathepsin-L inhibitor (data not shown). These observations indicate that the hypericin-enhanced HIF-1α turnover is mediated through catabolism by a cathepsin-B type enzyme independently of the ubiquitin-proteasome pathway.

Stable transfection of VHL into C2^VHL−/−^ cells restored the E3 ubiquitin ligase complex functionality [19], including HIF-1α proteasomal degradation under normoxia and HIF-1α stabilization under hypoxic conditions (Fig. 1D). In this setting hypericin also accelerated HIF-1α degradation under hypoxia, abrogating the hypoxia-induced HIF-1α stabilization in VHL-reconstituted RCC-C2^VHL+^ cells (C2^VHL+^ cells) (Fig. 1D). HIF-1α degradation by hypericin in C2^VHL+^ cells was also inhibited by CA-074 (Fig. 1D). Thus, the cathepsin-B pathway remains the major route of HIF-1α degradation by hypericin under hypoxia following pVHL reconstitution.

### Hypericin elicits reductions in intracellular pH

The shift in HIF-1α turnover from the physiologic pVHL-mediated proteasomal degradation to a cathepsin-B dependent mechanism caused by hypericin prompted us to investigate whether changes in intracellular conditions contributed to this shift. Since light-induced photodynamic activities of hypericin elicit reductions in the intracellular pH (pH_i_) of tumor cell [20], we hypothesized that hypericin can also cause intracellular pH reduction in the dark via redox activities, optimizing conditions for lysosomal enzyme activity. pH_i_ was measured in U87-MG and C2^VHL−/−^ cells treated with 10, and 30 µM hypericin for 72 hrs in the dark, monitoring pH dependent esterolytic cleavage of the acetoxymethyl-ester derivative of BCECF (please see Methods for details). Despite strict maintenance of dark conditions, we recorded hypericin concentration dependent reductions in intracellular pH in U87-MG and C2^VHL−/−^ cells (Fig. 1E). The cytosolic pH_i_ decreased in U87-MG cells from a baseline of 7.12±0.08 to 6.75±0.06 with 10 µM of hypericin (p≤0.05), and to 6.45±0.20 with 30 µM hypericin (p≤0.03). In C2^VHL−/−^ cells the pH_i_ declined from 7.18±0.04 to 6.85±0.28 (p≤0.08) with 10 µM hypericin and to 6.42±0.2 with 30 µM hypericin (p≤0.05). These studies show that hypericin also elicits concentration dependent reductions of cellular pH_i_ in the dark.

To determine whether reduced pH_i_ is essential for cathepsin-B mediated HIF-1α degradation by hypericin, effect of cytoplasmic alkalinization with NH_4_Cl on HIF-1α cytosolic content was examined in hypericin-treated cells. U87-MG cells were exposed to hypericin for 72 hrs in the dark and 150 µM CoCl_2_ hypoxia induced during the last 6 hrs of incubation in media supplemented with 20 mM ammonium chloride to elicit cytoplasmic alkalinization. Cytosolic extracts were prepared and Western blots developed with antibody to HIF-1α. Fig. 1F shows that prevention of intracellular pH reduction diminished HIF-1α degradation by hypericin, however at the higher 30 µM hypericin concentration some HIF-1α degradation under hypoxia did take place. This degradation appeared to be less sensitive to the cathepsin-B inhibitor CA-074 and may reflect involvement of a proteasome pathway dependent mechanism.

### Hypericin interferes with HIF-1α binding to VEGF and GLUT1 gene promoter HRE sequences

**Figure 2:**
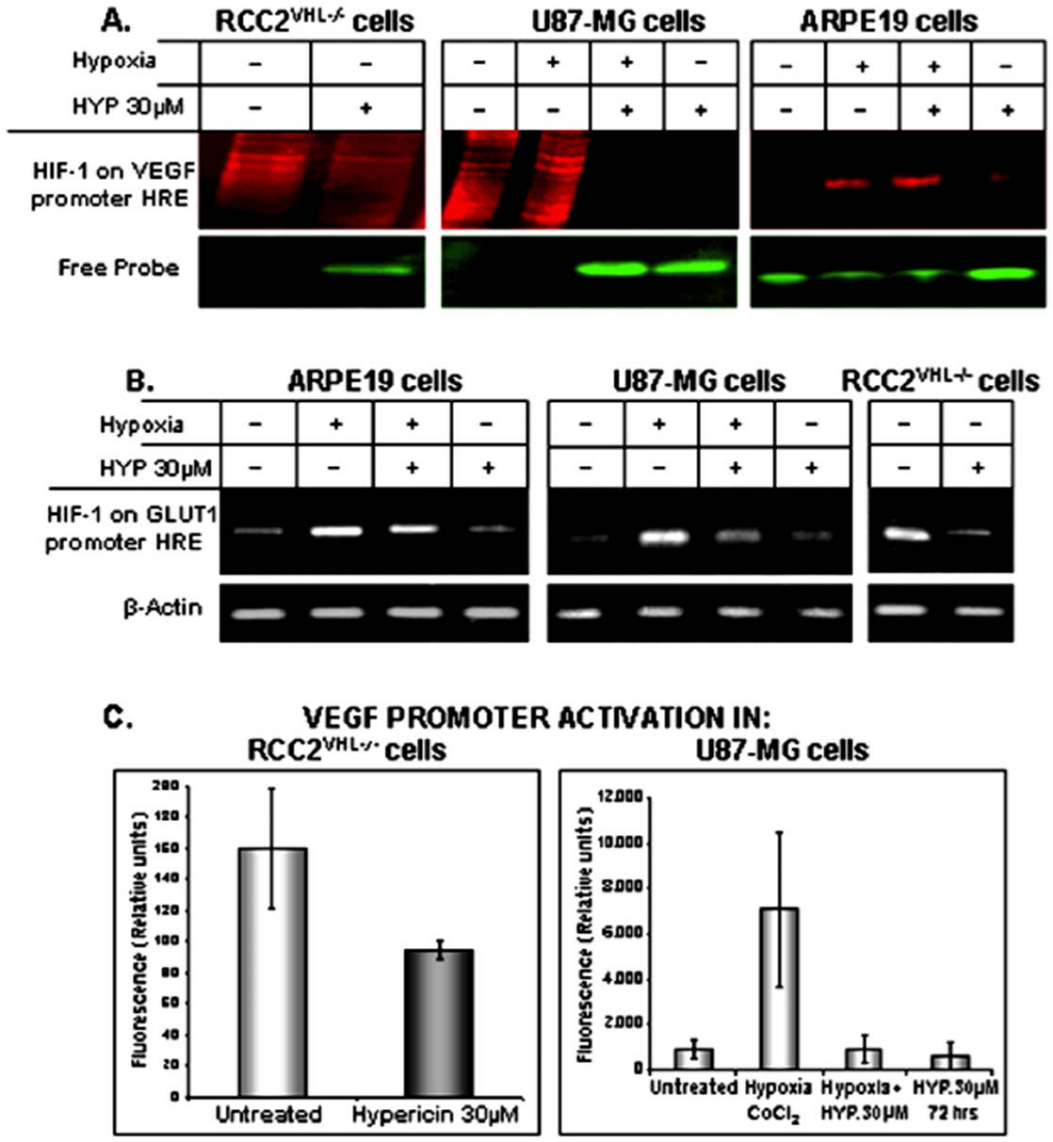
Interactions of HIF-1 with hypoxia response elements (HREs). Found on promoters of: [A]. The VEGF gene, analyzed by Fluorescence Electromobility Shift Assay (F-EMSA). Nuclear proteins were separated on 6% SDS-PAGE, stained with SYBER Green labeled DNA probe and unbound protein detected with SYPRO Ruby protein binding fluorochrome. Gels were scanned using FL-5000 laser-based scanner. [B]. The GLUT1 gene analyzed by chromatin immunoprecipitation (ChIP). Sheared chromatin was immunoprecipitated with anti HIF-1α antibody and DNA isolated from this chromatin was amplified with primers specific for the GLUT1 gene promoter region. [C]. Interference by hypericin with VEGF promoter activation in tumor cell lines. Left figure - C2^VHL−/−^ cells. Lane 1 – untreated cells, lane 2 cells treated with 30 µM hypericin for 72 hrs. Right figure - U87-MG cells. Lane 1 – untreated cells, lane 2 – cells subjected to hypoxia (150 µM CoCl_2_ for last 6 hrs), lane 3 - CoCl_2_-hypoxia for last 6 hrs and hypericin 30 µM for 72 hrs, and lane 4 - hypericin, 30 µM for 72 hrs.

The hypericin enhanced HIF-1α degradation and consequent HIF-1 nuclear content deficiency, were hypothesized to diminish HIF-1 interactions with hypoxia response elements (HREs) on stress response gene promoters as VEGF and GLUT1. HIF-1 binding to human VEGF gene promoter HRE was, therefore analyzed in hypericin-treated U87-MG, C2^VHL−/−^ and ARPE-19 cells using Fluorescence Electromobility Shift Assays (F-EMSA). Cells were exposed to hypericin for 72 hrs, nuclear extracts prepared and DNA-protein complex formation with VEGF promoter HRE element-containing fluorescent probes were analyzed for SYPRO Ruby Fluorescence. In C2^VHL−/−^ cells bearing high baseline HIF-1 content, HIF-1 formed DNA-protein complexes with the HRE probe (Fig. 2A). No free probe was detected (SYBER Green staining, left panel), reflecting a mostly bound DNA probe. Upon treatment with 30 µM hypericin, HIF-1-HRE complex formation was markedly decreased with most DNA probe remaining unbound (lane 2).

In normoxic U87-MG cells, HIF-1-HRE complex levels were low and increased under hypoxia leaving no detectable free DNA probe. Hypoxia-induced, HIF-1–HRE complex formation was abrogated by hypericin cell treatment (Fig. 2A, middle panel) leaving the DNA probe mostly unbound. In hypoxic ARPE-19 cells protein-HRE complex levels were higher compared to normoxic baselines and hypericin effectively reduced HIF-1 - probe binding. Similarly, free probe levels low in hypoxic cells, increased following exposure to hypericin (Fig. 2A, right panel). Treatment with hypericin of cell lines with high HIF-1 levels due to hypoxia or defective pVHL (C2^VHL−/−^ cells) resulted in reduced interactions with hypoxia response elements.

HIF-1 interactions with other stress response gene-promoter HREs as the GLUT1 gene were also affected by hypericin as shown by chromatin immunoprecipitation analyses. Chromatin prepared from nuclei of hypericin-treated and control cells was sheared and HIF-1 containing fragments immunoprecipitated with anti-HIF-1 antibody. HRE-bound HIF-1 levels were determined from amplification of extracted DNA by PCR using GLUT1 promoter specific probes. In U87-MG cells exposure to hypoxia resulted in increased HIF-1 interactions with GLUT1 promoter HRE (Fig. 2B middle panel), whereas concomitant exposure to hypericin reduced the amounts of promoter bound HIF-1 (lane 3). Similar reductions occurred in ARPE19 cells (Fig. 2B, left panel). In C2^VHL−/−^ cells, hypericin caused dramatic declines in GLUT1 promoter-bound HIF-1 (Fig. 2B, right panel). Overall, the reductions in HIF-1α cytoplasmic contents in hypericin-treated hypoxic cells also resulted in reduced mature HIF-1 transcription-promoting activities in the cell nuclei, diminishing HIF-1 interactions with the VEGF and GLUT1 gene promoters.

### Hypericin interferes with VEGF promoter activation in tumor cell lines

The decreases in HIF-1 binding to VEGF and GLUT1 promoters caused by hypericin, prompted analyses of the effects of hypericin on VEGF promoter activation. U87-MG and C2^VHL−/−^ cells were transiently transfected with a reporter construct, containing the HRE element of VEGF-A gene (pGL3P-1100). The cells were split into 30 µM hypericin treated group (for 72 hrs) and untreated control group. Luciferase activity was measured against the pGL3P vehicle control vector transfected cells devoid of the reporter construct. Fig. 2C (left exhibit) shows expression of high luciferase activity in untreated control C2^VHL−/−^ transfectant cells, which showed a trend towards suppressed luciferase activity in hypericin-treated C2^VHL−/−^ cells by approximately 40%, suggesting that hypericin tends to interfere with VEGF promoter activation in C2^VHL−/−^ cells, however the differences did not reach statistical significance (*P* = 0.06, Mann Whitney test). Luciferase levels of vehicle plasmid pGL3P were low and similar in both groups (not shown).

In U87-MG cells, the VEGF promoter was also strongly activated in response to hypoxia (Fig. 2C), whereas cell treatment with 30 µM hypericin completely abolished the hypoxia-induced promoter activation (*P* = 0.05) (3^rd^ column). Hypericin alone without hypoxia had no effect on promoter activation (4^th^ column). pGL3P vehicle control vector transfectants were low in all groups (not shown). Thus, hypericin can inhibit VEGF-promoter activation under hypoxic conditions via HIF-1α degradation in U87-MG cells.

### Hypericin modulates VEGF gene transcription in tumor cell lines

**Figure 3:**
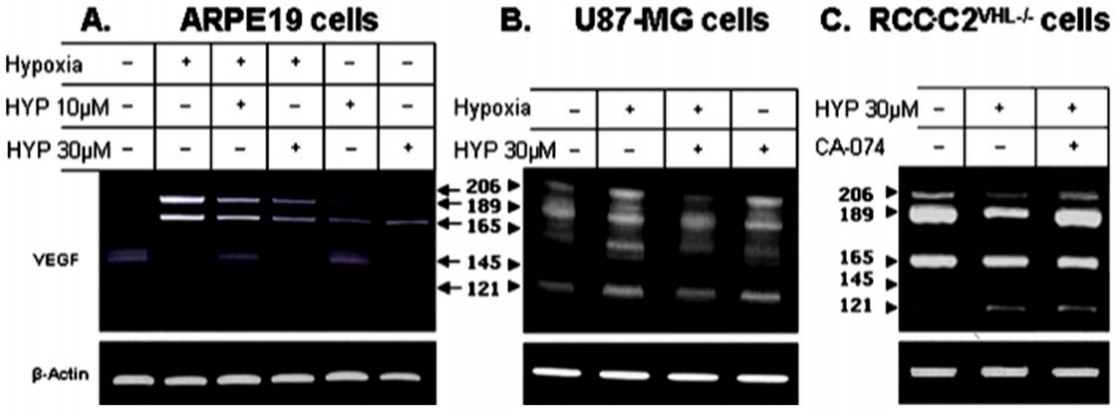
Modulation of expression patterns of the different VEGF-A splice variants by cell treatment with hypericin. A. In ARPE-19 cells, B. in U87-MG cells and C. in C2^VHL−/−^ cells.

The downregulation of VEGF promoter activation due to hypericin-induced HIF-1α catabolism in hypoxic cells prompted investigation of the effects of the hypericin-elicited, HIF-1α catabolism on VEGF gene transcription. U87-MG and ARPE-19 cells exposed to hypericin (72 hrs in the dark) were subjected to CoCl_2_ hypoxia for the last 6 treatment hours. RNA was prepared and VEGF transcription analyzed by semi-quantitative RT-PCR using primers spanning exon1 and exon8 of VEGF-A gene. These primers amplify all six VEGF splice variants and the semiquantitative analyses enable differential profile analyses of levels of each VEGF splice transcript. The results show that in ARPE-19 cells, hypoxia induced modulations in VEGF splice variant transcriptional profiles. When hypoxia was combined with exposure to hypericin, transcription of VEGF_189_ and VEGF_165_ isoforms which dramatically increased under hypoxia declined (Fig. 3A), particularly with the 30 µM dose (Fig. 3A, lane 4). VEGF_145_ expressed in untreated control cells and suppressed under hypoxia was re-expressed following hypericin treatment (Fig. 3A, lane 4).

In U87-MG cells baseline levels of some VEGF isoforms under normoxia, mainly VEGF_189_ were high due to hypoxia-independent mechanisms as reactive oxygen species [21], NO [22], phosphatidylinositol-3-kinase and MAP kinase activation through the action of mTOR [23], or loss of IDH1 gene function in glioblastoma [9]. Nevertheless transcription of VEGF isoforms primarily VEGF_206_, VEGF_165_ and VEGF_121_ increased following cell exposure to hypoxia. Their transcription decreased after cell exposure to 30 µM hypericin (Fig. 3B, lane 3). In C2^VHL−/−^ cells constitutively expressing HIF-1α, VEGF transcription was consequently very high at baseline. Exposure to hypericin (72 hrs) induced reductions in VEGF_206_, VEGF_189_ and marginally also VEGF_165_ (Fig. 3C). Interestingly, when hypericin-treated cells were also exposed to CA-074 (100 µg/ml), the cathepsin-B inhibitor which prevented the hypericin-enhanced HIF-1α degradation, VEGF gene transcription was also stimulated compared to cells treated with hypericin only (Fig. 3C). In summary, hypericin stimulated changes in VEGF splice variant expression patterns most notably in ARPE-19 cells (Fig. 3A) and also detectable in U87-MG and C2^VHL−/−^ cells.

### Prevention of VEGFR2/KDR expression in hypericin treated cells

The modulations in VEGF gene transcription patterns induced by hypericin-mediated accelerated HIF-1α degradation, warranted analyses of hypericin effects on mediators of angiogenesis at the protein level. Possible correlations with Hsp90 activities were also evaluated due to hsp90 polyubiquitination, inactivation and degradation which are also induced by hypericin [14], and the roles hsp90 plays in key sites of the angiogenic cascade [2], [24].

**Figure 4:**
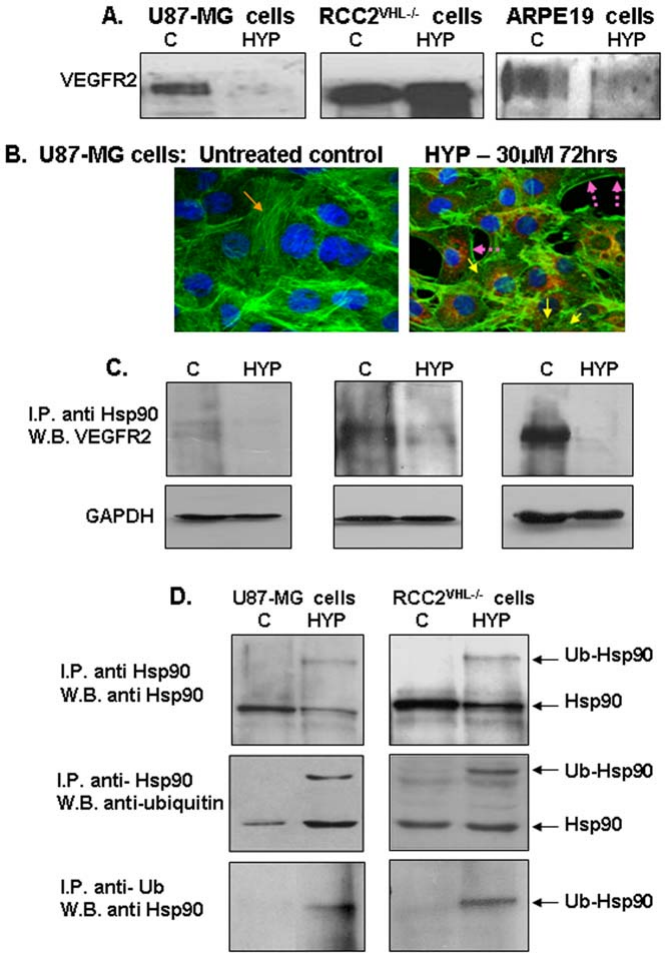
Effects of hypericin on complex formations between VEGFR2, Hsp90 and on HOP-hsp70 intermediate complexes. [A]. Western blot analyses of VEGFR2 protein levels in cytosolic extracts from untreated control cells (C), or cells treated with 30 µM hypericin for 72 hrs (HYP). [B]. Staining of U87-MG cells with Phalloidin-FITC. (1) Untreated cells and (2) cells treated with hypericin 30 µM for 72 hrs. Orange arrows show F-actin filaments; yellow arrows depict collapsed actin globules following exposure to hypericin. [C]. VEGFR2-hsp90 complex formation following treatment with hypericin 30 µM for 72 hrs. Results of immunoprecipitation with anti-Hsp90 antibody and development of Western blots with anti-VEGFR2 antibody. Hypericin diminished VEGFR2-Hsp90 complex formation. [D]. Induction of forced hsp90 poly-ubiquitination by hypericin (30 µM for 72 hrs) in human cancerous cell lines. Top panel – immunoprecipitation with anti-hsp90 and Western blot with anti-hsp90 antibody (control); middle panel - immunoprecipitation with anti-hsp90 and Western blot with anti-ubiquitin, and lower panel immunoprecipitation with anti-ubiquitin and Western blot with anti-hsp90. (I.P – immunoprecipitation; W.B. – Western blots).

Effects of hypericin treatment on VEGFR2/KDR expression were examined in the three cell lines, including ARPE-19 because VEGFR2 is also expressed on RPE cells [25]. The treatment with hypericin induced marked suppression of VEGFR2 cellular content in U87-MG and ARPE-19 cells however, receptor levels were unaffected in renal C2^VHL−/−^ cells (Fig. 4A). To determine if reduced VEGFR2 expression resulted from diminished VEGF production we supplemented the growth medium of U87-MG and ARPE-19 cells with VEGF (10 ng/ml) with or without heparin (1 unit/ml for 48 hrs) one day after hypericin administration and analyzed VEGFR2 expression in these cells. These treatments did not modify the hypericin downregulated VEGFR2 expression and did not increase VEGFR2 levels (data not shown). We therefore, examined whether hypericin modulated VEGFR2 mRNA expression levels in these cells. RNA was prepared from hypericin treated cells (72 hrs in the dark) and semiquantitative RT-PCR analyses performed with VEGFR2-specific primers, using VEGFR1 as comparative reference. These experiments revealed that VEGFR2 gene transcription was effectively and selectively inhibited by hypericin in all three cell lines, whereas VEGFR1 transcription was less affected (Figure S1). They suggest that hypericin triggers epigenetic effects selectively downregulating expression of VEGFR2 and several additional genes. However this large topic exceeds the scope of this manuscript and will be further discussed elsewhere.

VEGFR2 downstream signaling following activation by VEGF-VEGFR2 interactions also requires association with hsp90 [24]. Hsp90 involvement is relevant to our analyses of antiangiogenic effects, because we previously reported in murine breast and squamous carcinoma tumor cells that hypericin induces hsp90 polyubiquitination and accelerated degradation, destabilizing and degrading hsp90-client proteins in these cells [14]. VEGFR2 downstream signaling activates alphavbeta3 and focal adhesion kinase (FAK) to form cytoskeletal focal adhesions and F-actin polymers [24]. FAK and Src are also hsp90 client proteins and indeed hypericin treatment interferes with F-actin polymerization (Fig. 4B). We therefore investigated the hypericin effects on VEGFR2 association with hsp90, analyzing VEGFR2 pull down following immunoprecipitation with anti-hsp90 antibody. Fig. 4C shows that VEGFR2 co-immunoprecipitates with hsp90, however this co-immunoprecipitation was abrogated following treatment with 30 µM hypericin in all three cell lines. This apparently universal finding indicates that VEGF-VEGFR2-Hsp90 complex formation diminishes due to actions of hypericin.

We also confirm here that hypericin induces hsp90 polyubiquitination in human cell lines. Immunoprecipitation of U87-MG and C2^VHL−/−^ cell lysates with anti-hsp90 pulled down ubiquitin following hypericin exposure and vice versa: immunoprecipation with anti-ubiquitin pulled down hsp90 (Fig. 4D).

### Hsp90 poly-ubiquitination interferes with the nuclear transport of HIF-1α prior to degradation of HIF-1α

**Figure 5:**
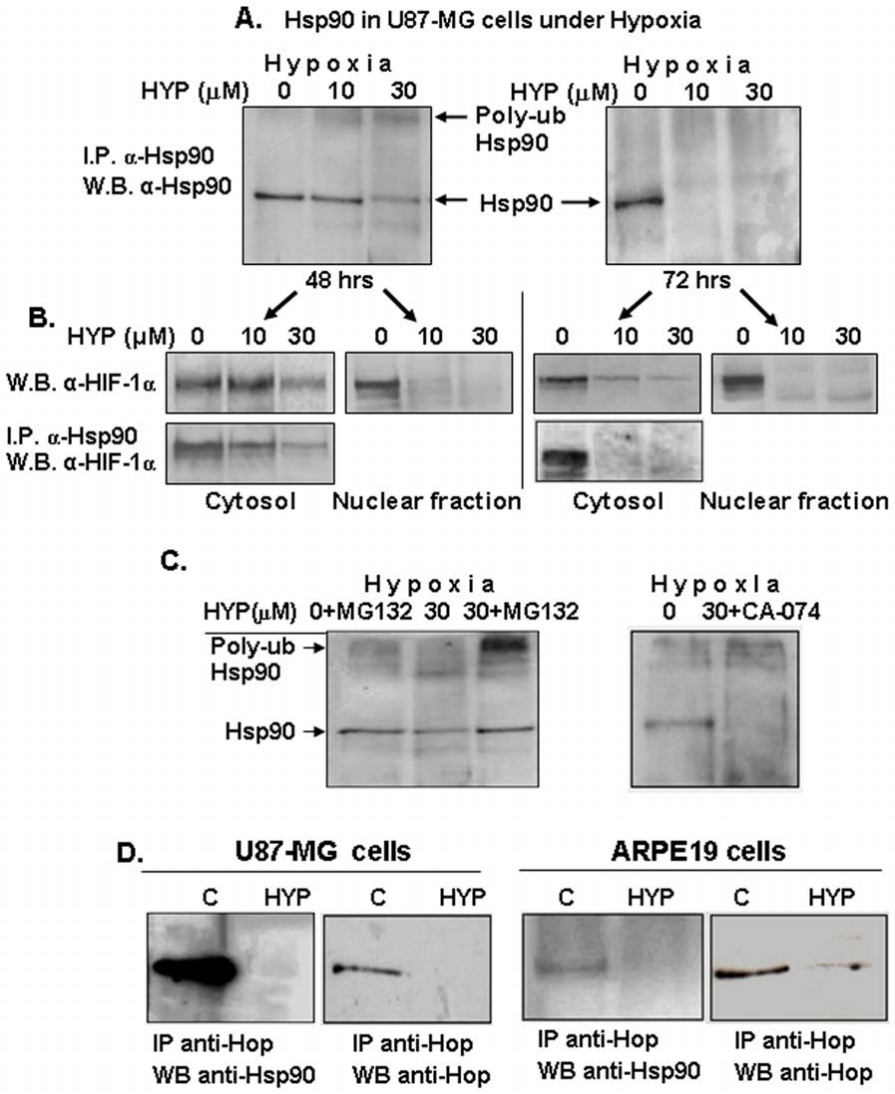
Effects of hypericin-mediated hsp90 poly-ubiquitination on the destabilization of HIF-1α in U87-MG cells under hypoxia. U87-MG cells were exposed to 10 & 30 µM hypericin for 48 hrs (left panels) and 72 hrs (right panels) under hypoxic conditions (150 µM CoCl_2_) and hsp90 expression and chaperone activities were analyzed. Cytosolic extracts were immunoprecipitated with anti-hsp90 and Western blots developed with: [A] anti-hsp90 antibodies. [B] anti HIF-1α. (lower panel), which analyzed hsp90-bound HIF-1α levels. Bound HIF-1α levels were compared to total HIF-1α obtained from Western blots performed on whole cytosolic (left panels) and nuclear extracts (right panels). (HYP denotes hypericin). [C]. Sensitivity of hypericin-induced poly-ubiquitinated Hsp90 to proteasome and cathepsin B inhibitors in U87-MG cells. Left panel – MG132 proteasome inhibitor and right panel CA-074, cathepsin B inhibitor. [D]. Effects of cell treatment with hypericin on formation of Hop co-chaperone-mediated hsp90-hsp70 intermediate complexes. Analyses of Hop content in these complexes: left pair refers to U87-MG cells and right pair to ARPE19 cells.

To determine whether the hypericin-mediated destabilization of HIF-1α also depends on the hypericin-induced poly-ubiquitination of hsp90 we performed time dependent evaluations of the effects of hypericin on hsp90 poly-ubiquitination and contemporarily on hsp90-associated HIF-1α cellular contents in U87-MG cells under hypoxia. The cells were exposed to 10 and 30 µM of hypericin for 48 hrs at which time hsp90 poly-ubiquitination became detectable and for 72 hrs when the chaperone has been degraded [14]. Hypoxia was induced with CoCl_2_ for the last 6 hours of the experiment, the cells lysed and both cytosolic and nuclear extracts were prepared. Hsp90 was immunoprecipitated with its corresponding antibody and Western blots developed with anti-HIF-1α antibodies to monitor for hsp-90 bound HIF-1α and with anti-hsp90 as control (Fig. 5A). HIF-1α levels bound to hsp90 were compared with total HIF-1α analyzed on Western blots directly from whole cytosolic and nuclear fractions. The results show that following exposure to hypericin for 48 hrs hsp90 became poly-ubiquitinated, yet chaperone degradation was primarily noted at the higher dose of 30 µM hypericin (Fig. 5A, left image). Cytosolic HIF-1α began to be degraded at the 48 hr time point, only after treatment with 30 µM hypericin (Fig. 5B, upper left panel). However, HIF-1α nuclear transport was most strongly abrogated by both hypericin dose levels (Fig. 5B, 48 hr time-point 2^nd^ panel, nuclear fraction). This was due to the high dependence of HIF-1α nuclear transport on intact hsp90 chaperone activity [26]. Hsp90 poly-ubiquitination by hypericin caused loss of chaperone activity and prevented the physiologic HIF-1α trans-migration into the nucleus where it normally dissociates from hsp90 [27]. HIF-1α bound to hsp90 and co-immunoprecipitated with anti hsp90 antibody was also more strongly degraded due to hsp90 ubiquitination after 48 hrs (Fig. 5B, lower left panel). At 72 hrs cytosolic hsp90 was highly degraded to below detection level (Fig. 5A, right image) and affected both the co-immunoprecipitated HIF-1α and HIF-1α transported into the nucleus (Fig. 5B, third and forth images). The total cytosolic HIF-1α content at the 72 hr time-point was also strongly degraded, yet some HIF-1α protein remained detectable. Thus, cytosolic HIF-1α associated with hsp90 decreased as poly-ubiquitination of hsp90 increased (Fig. 5A & 5B) but correlated less with the actual hsp90 degradation.

We hypothesized that these differences may be due to the disparity in mechanisms of hsp90 and HIF-1α degradation in hypericin treated cells: hsp90 via the ubiquitin-proteasome pathway and HIF-1α via cathepsin B (Fig. 1C). Since hypericin modulates the intracellular pHi (Fig. 1E) and the intracellular pHi affects HIF-1α degradation (Fig. 1F), we also examined whether turnover of ubiquitinated hsp90 under reduced intracellular pH conditions is mediated by the proteasome or is also affected by cathepsin B. U87-MG cells exposed to hypericin (72 hrs), were subjected to hypoxia, to the MG132 proteasome inhibitor and to CA-074, the cathepsin B inhibitor for the last 6 hrs of the experiments and effects on hsp90 levels were monitored. Fig. 5C, left panel shows that presence of MG132 prevented hsp90 degradation pointing to a major role for the proteasome in hypericin-induced, poly-ubiquitinated hsp90 degradation, however, unlike HIF-1α, hsp90 remained unaffected by CA-074 (Fig. 5C, right panel). Although HIF-1α is tightly bound by hsp90 in the cell cytosol, the two dissociate following hsp90 ubiquitination and undergo degradation via different cellular protein turnover pathways.

The ubiquitination of hsp90 by hypericin also affects activities of hsp70 chaperones. Intermediate complexes these two chaperones form are regulated by a cohort of co-chaperones, enabling hsp90 to affect hsp70 client proteins that together regulate folding of large numbers of cellular proteins. We examined possible implications of hsp90 ubiquitination by hypericin on Hsp70/Hsp90 intermediate complex formation, by analyzing cell contents of the important stress-inducible protein 1 (STI1) co-chaperone known as the “Hsp70/Hsp90 Organizing Protein” (HOP). HOP associates with both Hsp70 and Hsp90, thereby regulating their activities [28]. Fig. 5D shows that destabilizing hsp90 by hypericin abrogated hsp90-Hop intermediate complex formations, preventing their co-immunoprecipitation. Furthermore, the hsp90 inactivation and degradation caused dramatic decreases in Hop cellular contents. Similar observations were reported in human colon carcinoma cells in which HOP levels correlated with those of hsp90 [29]. These findings indicate that hsp90 poly-ubiquitination and chaperone inactivation caused by treatment of human tumorigenic cell lines with hypericin also eliminate VEGF-VEGFR2-Hsp90 complex formation as well as Hsp70-Hsp90-HOP intermediate complexes in cancerous cells.

## Discussion

One example for a multi-targeting molecule which can inhibit tumorigenesis [30], angiogenesis [13] and development of metastases [12], is hypericin. To exert this anticancer activity array, hypericin must affect several key processes at crossroads controlling multiple cell signaling pathways. Our initial studies identified hsp90 as one major hypericin target, being the sole exogenous reagent known to selectively bind to and induce forced hsp90 poly-ubiquitination [14]. Hsp90 poly-ubiquitination is disparate from the negative regulatory hsp90 mono-ubiquitination which follows hsp90 hyper-acetylation due to suppressed HDAC6 activity [31]. HDAC6 has hsp90 among its target substrates [32]–[33]. Unlike this reversible mono-ubiquitination, hypericin-forced hsp90 poly-ubiquitination accelerates degradation of this chaperone causing loss of its activities [14]. Hsp90 client proteins, many with signaling kinase activities, are consequently destabilized and rapidly degraded, generating deficiencies in key signaling mediators compromising pathways vital for angiogenesis, cell migration and invasion. We initially observed this phenomenon in murine tumor cells [14] and confirm here its occurrence in human brain and kidney cancer cell lines (Fig. 4D). We also show here that hsp90 poly-ubiquitinated by the effects of hypericin is degraded in the proteasome (Fig. 5C). Vital co-chaperones as HOP are additional targets that become degraded following hsp90 poly-ubiquitination, potentially affecting hsp70 client proteins as well.

The stress-response transcription factor precursor HIF-1α is another key signaling regulator shown here to be targeted by hypericin for accelerated degradation. HIF-1α also depends on hsp90 for stability and maturation into HIF-1 and is degraded by geldanamycin derivatives [34]. The hallmark of the HIF-1α response to stress and hypoxia includes HIF-1α protein stabilization [17], [35], its activation and nuclear translocation. These are abrogated by hypericin which triggers accelerated HIF-1α degradation via a unique mechanism which is independent of cellular oxygen levels. Hypericin is photodynamic, possessing unique electron accepting and donating properties that enable action as both oxidizing and reducing agent [36]. Electrochemical and electron paramagnetic resonance studies suggest that its redox potential in physiological pH (E_1_/V = −1.01 mEV) is moderately lower than those of cellular bioenergized electron transfer reaction mediators. Thus, hypericin can scavenge electrons to propel its redox activities within cells in the dark [37].

The hypericin-enhanced HIF-1α degradation which follows the poly-ubiquitination of hsp90 and loss of activity of this chaperone, overcomes the hypoxic HIF-1α stabilization generated either by CoCl_2_ chemical hypoxia or by a low oxygen atmosphere (Figs. 1A & 1B). It can, therefore occur in cells in which normal oxygen-dependent HIF-1α degradation is disrupted as the VHL deleted C2^VHL−/−^ cells (Fig. 1C). The resulting high constitutive HIF-1α intracellular accumulation generates highly vascularized tumors as renal cell carcinoma or hemangioblastoma. The degradation of HIF-1α in C2^VHL−/−^ cells with hypericin reverses the high constitutive HIF-1α cellular accumulations in manners that are independent of the proteasome and unaffected by its inhibitors, yet sensitive to CA-074, a cathepsin-B inhibitor. Sensitivity to CA-074 also characterizes other tumor cell lines indicating preference for cathepsin-B in hypericin mediated HIF-1α turnover. Cathepsin-B activity may be optimized by hypericin-mediated reductions in intracellular pH_i_. Hypericin is known to cause light-dependent photodynamic intracellular pH_i_ reductions [20]; it is shown here that pH_i_ reductions also occur in the dark via the redox reactivities of hypericin. Under these conditions the hypericin-induced poly-ubiquitination of hsp90 disrupts the binding of hsp90 to client proteins as HIF-1α and accelerates the degradation of both hsp90 and HIF-1α, however hsp90 turnover occurs in the proteasome (Fig. 5C) whereas HIF-1α is degraded by cathepsin B (Fig. 1C).

The effects on HIF-1α classify hypericin among HIF-1α degradation-enhancing agents as the HDAC inhibitor Trichostatin A [38] and 17-Allylaminogeldanamycin (17-AAG) [34], although the mechanism of the hypericin action may be different. Nevertheless, since hsp90 is required for HIF-1α transport into the cell nucleus [2], hsp90 inactivation by hypericin may also accelerate HIF-1α degradation similarly to 17-AAG [15]. Indeed, hypericin also interferes with HIF-1α migration into the cell nucleus preventing its heterodimerization into a mature transcription factor by creating deficiency in HIF-1α cytosolic content and by inactivating hsp90 required for HIF-1α nuclear migration. Consequently, HIF-1 binding to stress-response gene promoter HRE elements diminishes, suppressing their trans-activation and gene expression (Fig. 2).

Preliminary findings point to induction of epigenetic modulatory effects by cell treatment with hypericin, which future studies should address. These were evident in the highly effective downregulated expression of the VEGFR2 gene in all the three cell lines that were evaluated in this study, demonstrated in Figure S1. Interestingly, gene expression profiling studies revealed epigenetic modulations of gene expression patterns induced by another hsp90 inhibitor, the benzoquinone ansamycin, geldanamycin analogue, 17-AAG [43], which is also a HIF-1α degradation-enhancing agent [34].

The hypericin-induced HIF-1α degradation is likely to attribute important clinical utilizations to hypericin in management of pathological consequences of excessive responses to stress. Targeting HIF-1α for degradation is currently a vital option in anti-cancer therapy to inhibit tumor neoangiogenesis and glucose transport. Our findings suggest that such activities can be countered by hypericin.

Indeed, a Phase I/II clinical trial testing hypericin in patients with recurrent progressive glioblastoma multiforme (grade IV) and anaplastic astrocytoma (grade III) who failed other therapies, has been completed in 6 North American Medical Centers with promising outcomes. 7/35 GBM patients were objective responders (20%) with a median survival of 6 months, despite this difficult patient group. [44]. It should encourage further evaluation of hypericin in brain tumors and other cancer types in which high HIF-1α levels cause clinical exacerbation.

## Materials and Methods

### Preparation of hypericin

Hypericin (10,11-dimethyl-1,3,4,6,8,13 hexahydroxy naphthodianthrone) was synthesized from emodin according to a method developed by Y. Mazur. Emodin was converted to emodin anthrone by reflux in SnCl_2_^.^2H_2_O in HOAc, followed by reflux in concentrated HCl. The pellet was filtered, water-washed and dried at 40–50°C under vacuum to yield emodin anthrone. Self condensation of emodin anthrone to protohypericin was achieved by dissolving emodin anthrone in pyridine and heating with piperidine, pyridine-N-oxide and catalytic amounts of FeSO_4_ (Sigma-Aldrich Chemicals). This resulted in the formation of protohypericin.

Photoactivated ring closure of protohypericin to form hypericin was achieved by irradiation of protohypericin with visible light to yield free hypericin which was crystallized from pyridine resulting in a hypericin-pyridine complex. This complex was heated to 160°C for 2 hrs in high vacuum. The resulting free hypericin was dissolved in methanol and converted to a hypericin-monosodium salt by adding aqueous NaHCO_3_, precipitation with hexane and crystallization from methanol. Hypericin was purified to 98.7% by chromatographies on silica gel (Merck 60; 70–230 mesh) and eluted with methanol∶EtOAc 2∶1 and 1% aqueous NaH_2_PO_4_. For the bioassays hypericin was dissolved to 4 mM in 70% aqueous ethanol. Subsequent dilutions were made in culture media limiting the final concentration of EtOH to ≤1%.

Since hypericin is a potent photosensitizer all studies were conducted in strict darkness (ambient light maintained <300 mW/cm^2^).

### Cell lines and culture conditions

ARPE-19, a non-malignant human retinal pigment epithelial cell line, which expresses functional VEGF receptors [39] and U87-MG human glioblastoma cell line were obtained from the American Type Culture Collection. RCC2^VHL−/−^ human renal cell carcinoma cell line (VHL locus deleted) was a gift from Dr. W. Marston Linehan, NIH.

ARPE-19 cells were cultured in 1∶1 DMEM/F-12 nutrient medium, U87-MG and RCC-C2^VHL−/−^ in DMEM. All media were supplemented with 10% FBS, L-glutamine (2 mM), penicillin 100 U/ml and streptomycin 100 µg/ml (GibcoBRL, Paisley, Scotland).

### Chemicals

2′,7′-bis-(2-carboxyethyl)-5-(and-6)-carboxyfluorescein acetoxymethyl ester (BCECF), CoCl_2_, MG-132 and CA-074 were purchased from Sigma-Calbiochem (Darmstadt, Germany). N-Acetyl-Leu-Leu-Nle-CHO (ALLN) was from BIOMOL International LP.

### Hypoxia

Hypoxia was induced chemically using CoCl_2_ (150 µM), administered for the last 6 hrs of experiments and incubated in 37°C, 5% CO_2_ incubator for 6 hrs. In some experiments hypoxia was also generated by forming a low oxygen atmosphere of 94.5% N_2_, 5% CO_2_ and 0.5% O_2_.

### Preparation of nuclear and cytosolic cellular fractions

Nuclear and cytosolic extracts were prepared from U87-MG, RCC^VHL−/−^ and ARPE-19 cells. The cells were washed twice with PBS and swollen for 15 minutes on ice with buffer A consisting of 10 mM Hepes pH 7.9, 10 mM KCl, 1 mM EDTA, 1 mM EGTA, 1 mM DTT and complete protease inhibitor cocktail 40 µg/ml (Roche Molecular Biochemicals, Mannheim, Germany). NP-40 was then added to a concentration of 0.5% for induction of cell lysis and the nuclei sedimented by centrifugation at 14,000 g for 15 minutes at 4°C. The supernatants were collected for use as cytosolic fractions and stored at −70°C. Nuclear extracts were prepared by dissolution of nuclei in Buffer C containing 20 mM Hepes pH 7.9, 500 mM KCl, 1 mM EDTA, 1 mM EGTA, 1 mM DTT and complete Protease Inhibitor Cocktail, 40 µg/ml at 4°C for 15 minutes following centrifugation at 14,000 g for 10 minutes at 4°C. The purity of the cytosolic and nuclear fractions which were obtained using this method is demonstrated in Figure S2, which shows Western blots (left image) and Coomassie blue staining (right image) of cytosolic fractions (left 4 lanes) and nuclear fractions (right 4 lanes) separated on the same SDS-polyacryl-amide gels.

### Immunoprecipitation, Western Blot analyses

Cells were lysed in 10 mM HEPES pH 7.9, 10 mM KCl, 1 mM EDTA, 1 mM EGTA, 1 mM DTT buffer and 40 µg/ml Complete Protease Inhibitor mix (from Boehringer Mannheim, Germany). Immunoprecipitates were prepared using rabbit polyclonal anti-ubiquitin, anti-HIF-1α, anti-VEGFR2, anti-Hsp90 and anti-GAPDH antibodies (Santa Cruz Biotechnology, CA). Immune complexes were immobilized on protein A/G agarose beads (Santa Cruz, USA) and separated by SDS PAGE. Western blots were prepared using semi-dry transfer to nitrocellulose membranes following standard procedures, stained with peroxidase-tagged secondary antibodies and developed for chemiluminescence (SuperSignal® West Pico, Pierce, Rockford IL USA).

### Chromatin immunoprecipitation (ChIP)

DNA from 10^7^ cells/plate in 100 mm culture dishes was crosslinked to proteins with 1% formaldehyde in media for 10 min. Cells were washed ×2 with cold PBS containing 1 mM PMSF, 1 µg/ml aprotinin and 1 µg/ml pepstatin A, lysed in 200 µl SDS lysis buffer (1% SDS, 10 mM EDTA 40 µg/ml protease inhibitors mix, 50 mM Tris pH 8.1) and subjected to 5–10 sonication rounds (30 sec pulses at medium intensity). Cell debris were removed by centrifugation, the supernatants diluted 10 fold in ChIP dilution buffer (0.01% SDS, 1.1 Triton X-100, 1.2 mM EDTA, 16.7 mM Tris-HCl pH 8.1 and 167 mM NaCl), incubated overnight (4°C) with anti HIF-1α antibody and with salmon sperm DNA/protein A-agarose to reduce non-specific background. Pellets were washed with low salt, high salt buffer series (LiCl buffer and TRIS-EDTA [TE] buffer). DNA/protein complexes were eluted with 1% SDS, 0.1 M NaHCO_3_. DNA-protein crosslinks were reversed with 5 M NaCl at 65°C overnight. Immunoprecipitated DNA segments were purified with High Pure PCR Product Purification Kit; (Roche Diagnostics, Mannheim, Germany) and amplified by PCR using primers spanning the DNA binding sites of the immunoprecipitated protein. DNA fragments containing the HRE found on the human VEGF-A promoter were amplified using the primers:

F- 5′-CAGGAACAAGGGCCTCTGTCT-3′ and R- 5′–GCACTGTGGAGTCTGGCAAA–3′

### Intracellular pH measurements

Intracellular pH (pH_i_) was measured as pH-dependent decreases in fluorescence emission intensity of 2′,7′-bis-(2-carboxyethyl)-5-(and-6)-carboxy-fluorescein (BCECF), accumulating in cells following esterolytic cleavage of acetoxymethyl-ester derivative of BCECF (2′,7′-bis-(2-carboxyethyl)-5-(and-6)-carboxyfluorescein acetoxymethyl ester) by cellular esterases [40]. Fluorescence was measured using an Ascent Fluoroscan (ThermoLabsystems). Cells, 5×10^4^/well in 96 well flat bottom microplates were administered with hypericin and incubated for72 hrs in the dark. The growth medium was removed, the cells loaded with 0.33 or 1 µM BCECF-AM in PBS for 30 minutes at 37°C and unincorporated dye washed with PBS. Intracellular BCECF was excited at 506/460 nm and fluorescence emission measured at 485 nm. pH_i_ calibration to convert fluorescence intensity into pH values was achieved using the nigericin-K^+^ method, following exposure to high K^+^ buffer (140 mM KCl, 1.2 mM MgSO_4_, 1.2 mM KH_2_PO_4_, 1.3 mM CaCl_2_, 12.0 mM D-glucose, 10 mM HEPES, pH 6.0, pH 7.0 and pH 8.0 in the presence of 20 µM nigericin, a H^+^/K^+^ exchanger [41]. Cytoplasmic alkalinization to neutralize hypericin-induced reductions in intracellular pH was achieved by cell exposure to 20 mM NH_4_Cl [42].

### RT-PCR analyses

RNA was extracted in Trizol (Invitrogen, USA), washed with isopropanol and with ethanol air dried and dissolved in DEPC ddH_2_O. RNA content was calibrated spectrophotometrically and stored at −70°C. RNA OD_280_/OD_260_≥1.5 was used for amplification and semiquantitative RT-PCR analyses, performed using ReddyMix Reverse-iT kit (ABgene, UK).

Specific primers were designed from GeneBank™ sequences using the Primer3 Software (Whitehead Institute., USA) or derived from the literature. Amplified cDNAs were subjected to electrophoresis in 2% agarose gels containing 100 ng/ml ethidium bromide and photographed under UV light-induced fluorescence.

VEGF primers spanning Exon1 and Exon8 used were:

(F) 5′-TGCCTTGCTGCTCTACCTCC-3′),

(R) 5′- TCACCGCCTCGGCTTGTCAC-3′)

GLUT1 promoter region (SLC2A1 gene) (NM-006516):

(F) 5′–CTTCACTGTCGTGTCGCTGT–3′,

(R) 5′–TGAAGAGTTCAGCCACGATG–3′

VEGFR2

(F) 5′–GTGACCAACATGGAGTCGTG–3′,

(R) 5′–TGCTTCACAGAAGACCATGC–3′

VEGFR1

(F) 5′–GGCTCTGTGGAAAGTTCAGC–3′

(R) 5′–GCTCACACTGCTCATCCAAA–3′

β-Actin

(F) 5′–CATTGCTCCTCCTGAGCG–3′,

(R) 5′–CAACCGACTGCTGTCACC–3′.

### Transient transfection and luciferase reporter assay

Transient transfections of luciferase reporter plasmid pGL3-1100 containing the VEGF gene promoter HRE, driven by the SV-40 promoter (a gift from Dr. Casey C. Case, Sangamo Biosciences, Richmond, CA) were performed using Lipofectamine 2000 (Invitrogen, Carlsbad, CA). Empty pGL3 vector was used as mock transfection control (also obtained from Dr. Casey C. Case). Transfection efficiency was evaluated by co-tranfecting the two vectors with pGFP (green fluorescence protein plasmid) (and percent transfected cells monitored). Luciferase activity was evaluated using the Dual-Luciferase Reporter assay system (Promega, Madison WI) according to the manufacturer's instructions.

### Fluorescence electromobility shift assays (F-EMSA)

Nuclear extracts were prepared from test cells, calibrated and stored at −70°C. Samples were mixed with a DNA probe complementary to HRE sequences in binding buffer (750 mM KCl, 0.5 mM DTT, 0.5 mM EDTA, 50 mM Tris pH 7.4) for 15 min at 2°C. Samples were separated on 6% non-denaturing polyacrylamide gels by electrophoresis. Gels were stained with SYBER Green EMSA stain (which binds and stains DNA), (Molecular Probes, USA) for 20 min with gentle agitation followed by 2× washing with dH_2_O. DNA was visualized using FLA-5000 laser-based scanner, (Fujifilm Corp. USA) with filter which excites at 450 nm (or 488 nm) and emits at 520 nm. Thereafter, gels were stained with SYPRO Ruby EMSA stain for 3 hrs in the dark, (which binds and labels proteins), (Molecular Probes, USA), washed 2× in dH_2_O and scanned in laser-based FL5000 scanner using SYPRO Ruby filters (emission at 610 nm according to manufacturer's protocol).

## Supporting Information

Figure S1 **Selective downregulation of VEGFR2 gene expression by hypericin compared to VEGFR1.** RNA was prepared from the human cell lines, ARPE19, U87-MG and C2^VHL−/−^ after treatment with hypericin (72 hrs in the dark) and VEGFR2 mRNA expression was monitored compared to that of VEGFR1. Left lane untreated control cells, right lane treatment with hypericin (30 µM). Top panel VEGFR2 gene, middle panel VEGFR1 gene, bottom panel β-Actin housekeeping gene. Semiquantitative RT-PCR analyses. Hypericin induced strong downregulation of VEGFR2 gene transcription to below detection levels of the assay in all three cell lines. The effect was specific to VEGFR2 whereas VEGFR1 transcription was only marginally reduced in U87-MG and C2^VHL−/−^ cells and unaffected in ARPE19 cells.
(TIF)

Figure S2 **Comparisons of the protein profiles of cytosolic preparations with those of nuclear extracts prepared from the same cells.** Figure is presented for the sole purpose of validating the purity of each cellular fraction in preparations derived from U87-MG GBM cells (left exhibit) and RCC2^VHL−/−^ cells (right exhibit, Coomassie blue staining).
(TIF)
