# Supplementary figures and images for: Degradation of HIF-1alpha under Hypoxia Combined with Induction of Hsp90 Polyubiquitination in Cancer Cells by Hypericin: a Unique Cancer Therapy
Source: PLoS One. 2011 Sep 19;6(9):e22849. doi: 10.1371/journal.pone.0022849 (PMC3176203; doi:10.1371/journal.pone.0022849)

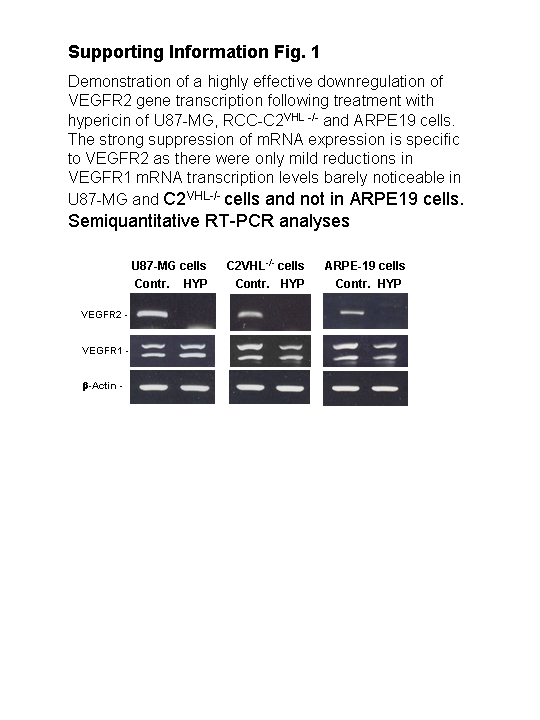

Supplement: Figure S1 — Selective downregulation of VEGFR2 gene expression by hypericin compared to VEGFR1. RNA was prepared from the human cell lines, ARPE19, U87-MG and C2VHL−/− after treatment with hypericin (72 hrs in the dark) and VEGFR2 mRNA expression was monitored compared to that of VEGFR1. Left lane untreated control cells, right lane treatment with hypericin (30 µM). Top panel VEGFR2 gene, middle panel VEGFR1 gene, bottom panel β-Actin housekeeping gene. Semiquantitative RT-PCR analyses. Hypericin induced strong downregulation of VEGFR2 gene transcription to below detection levels of the assay in all three cell lines. The effect was specific to VEGFR2 whereas VEGFR1 transcription was only marginally reduced in U87-MG and C2VHL−/− cells and unaffected in ARPE19 cells. (TIF) [file pone.0022849.s001.tif]

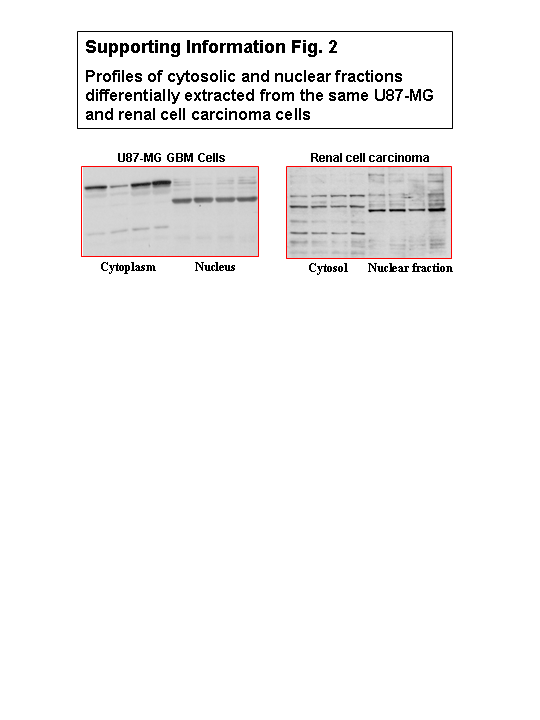

Supplement: Figure S2 — Comparisons of the protein profiles of cytosolic preparations with those of nuclear extracts prepared from the same cells. Figure is presented for the sole purpose of validating the purity of each cellular fraction in preparations derived from U87-MG GBM cells (left exhibit) and RCC2VHL−/− cells (right exhibit, Coomassie blue staining). (TIF) [file pone.0022849.s002.tif]
